# Supplementary material for: Agonistic CD27 antibody potency is determined by epitope-dependent receptor clustering augmented through Fc-engineering
Source: Commun Biol. 2022 Mar 14;5:229. doi: 10.1038/s42003-022-03182-6 (PMC8921514; doi:10.1038/s42003-022-03182-6)
Supplement: Supplementary file 2 — Supplementary information [file 42003_2022_3182_MOESM2_ESM.pdf]

Supplementary information

Agonistic CD27 antibody potency is determined by epitope-dependent receptor clustering augmented through Fc-engineering

Franziska Heckel[1,2], Anna H. Turaj[1,2], Hayden Fisher[1,2,3,4], H.T. Claude Chan[1,2], Michael J.E. Marshall[1,2], Osman Dadas[1,2], Christine A. Penfold[1,2], Tatyana Inzhelevskaya[1,2], C. Ian Mockridge[1,2], Diego Alvarado[5], Ivo Tews[3,4], Tibor Keler[5], Stephen A. Beers[1,2], Mark S. Cragg[1,2], Sean H. Lim[1,2,6,*]

[1]Centre for Cancer Immunology, Faculty of Medicine, University of Southampton, Southampton, SO16 6YD, UK

[2]Cancer Research UK Research Centre, Faculty of Medicine, University of Southampton, Southampton, SO16 6YD, UK

[3]Institute for Life Sciences, University of Southampton, Highfield Campus, Southampton, SO17 1BJ, UK

[4]Biological Sciences, University of Southampton, Highfield Campus, Southampton, SO17 1BJ, UK

[5]Celldex Therapeutics, Inc., Hampton, NJ 08827, USA

[6]Lead Contact

*Correspondence: s.h.lim@soton.ac.uk

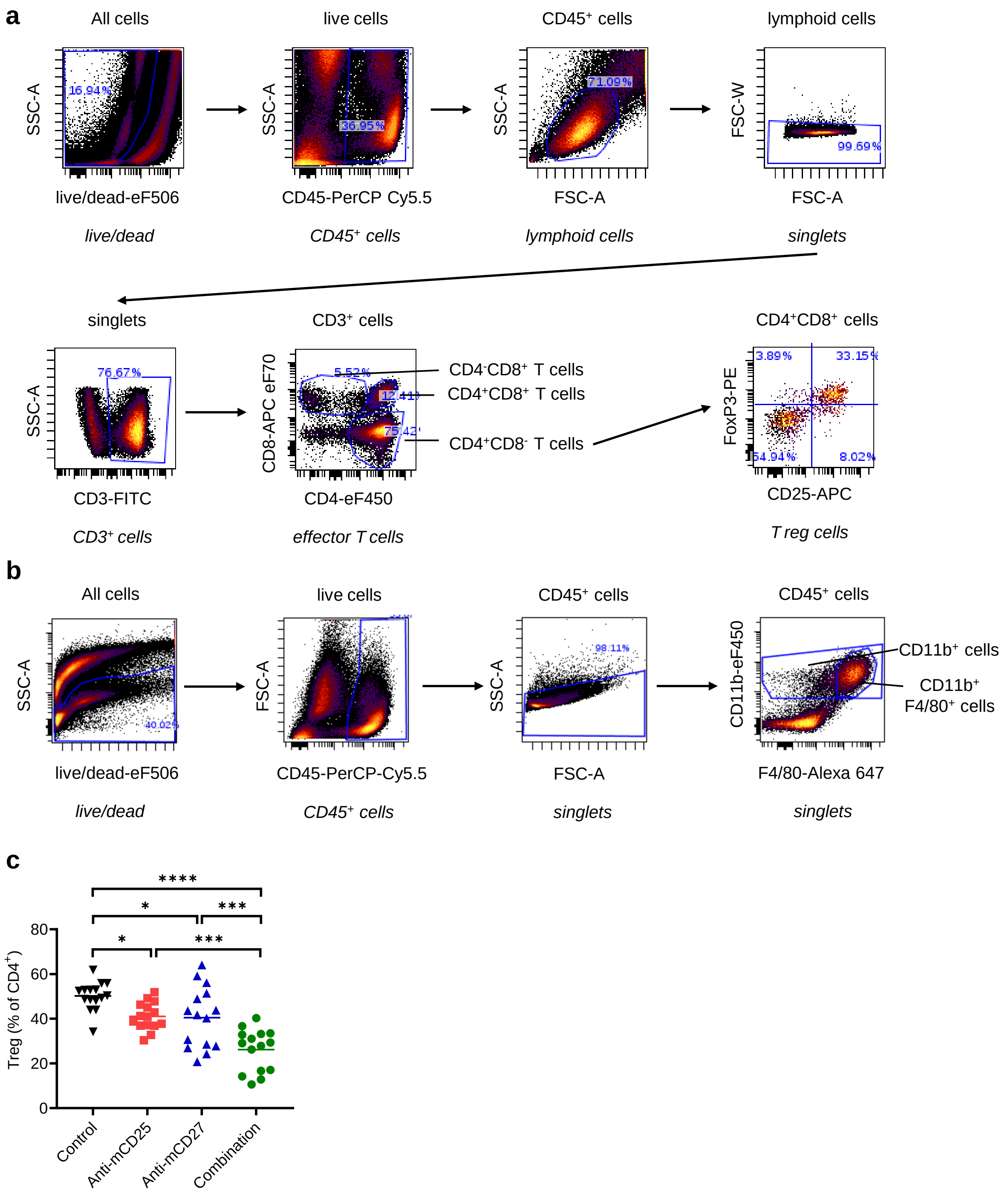

Supplementary Figure 1. CD27 stimulation enhances Treg depletion in CT26. CT26-bearing BALB/c mice were treated as described in Fig. 1a. Tumours were harvested on day 20 and analysed for immune cell subsets using flow cytometry. **a** Gating strategy for CD4+ and CD8+ T cells and Tregs. **b** Gating strategy for F4/80 on CD11b+ cells. **c** CT26-bearing mice were treated with either anti-mCD27 m1 (AT124-1, 100 µg) on days 11, 13, 16 and 18, anti-mCD25 r1 (PC61, 100 µg) on days 10 and 15 or the combination or the respective irrelevant isotype control and tumours were harvested on day 20. Shown is the percentage of tumour-infiltrating Tregs. Graph shows data from three independent experiments with n=15 per group. Data were assessed using one-way ANOVA and Tukey's test; *p <0.05, ***p < 0.001, ****p < 0.0001.

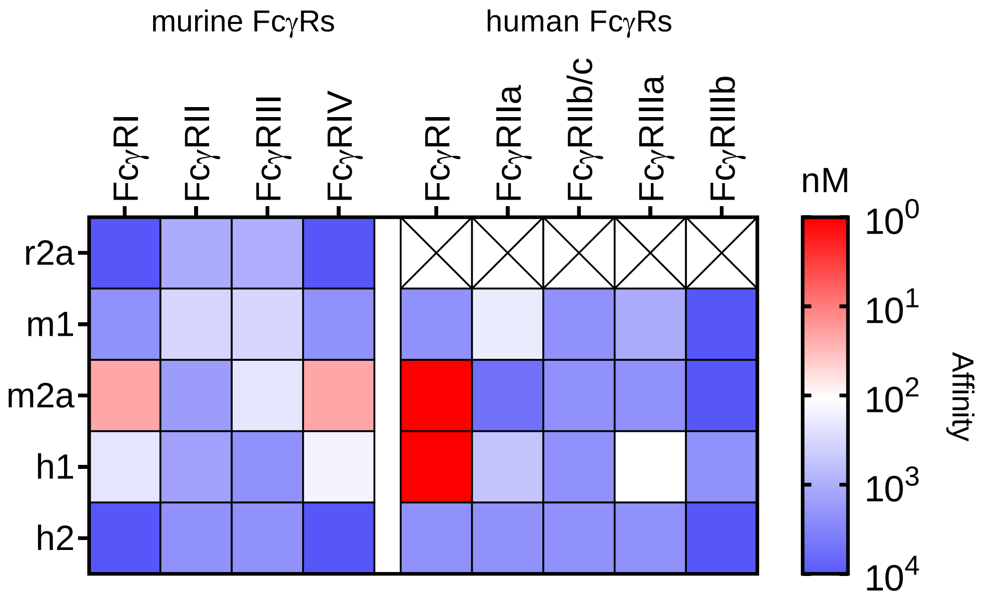

Supplementary Figure 2. Binding of rat, mouse and human mAb isotypes to murine and human FcγR determined by SPR. mAb (anti-CD40, anti-CD27, anti-CD20, anti-4-1BB, anti-OX40) of various isotypes (r2a, m1, m2a, h1, h2) were immobilised onto a SPR chip and murine or human FcγR injected to assess binding using a Biacore system. Red represents high affinity and blue low affinity of human and mouse FcγR to rat, mouse and human isotypes. X: Data was not assessed.

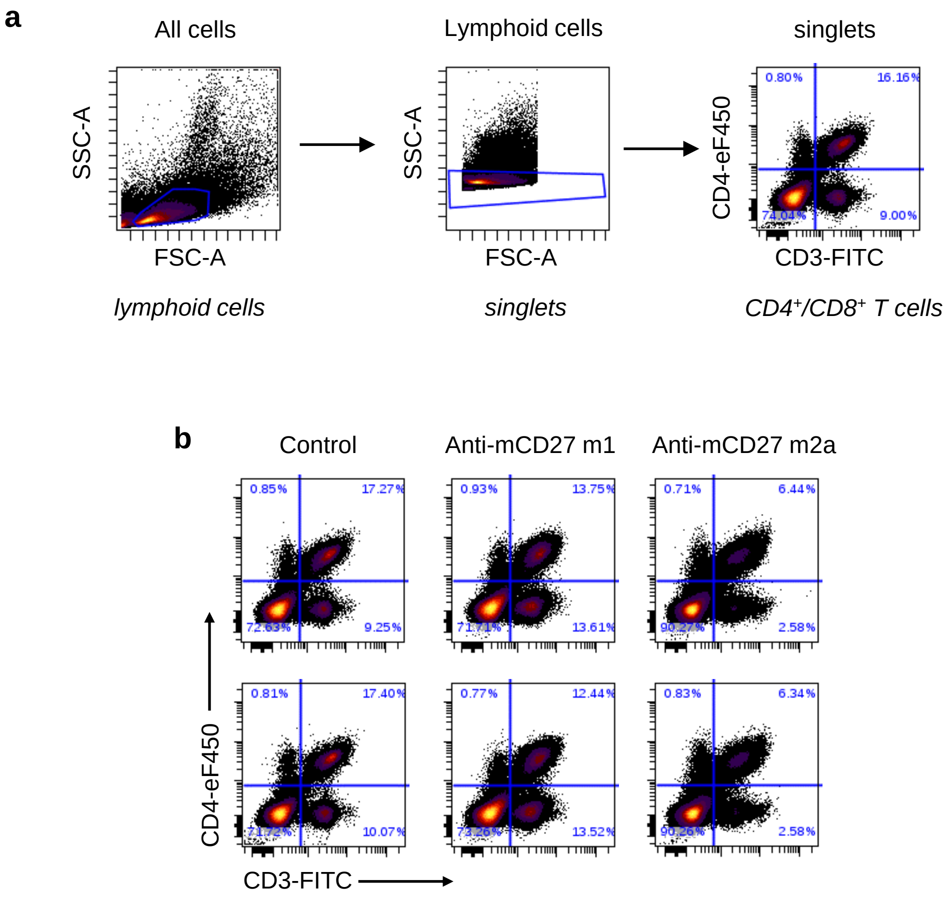

Supplementary Figure 3. Gating strategy of immune cell subsets in tumours of anti-CD27 treated BCL_1-bearing mice. a, b BCL_1-bearing BALB/c mice were treated as described in Fig. 2c. (**a**) Gating strategy for $CD4^+$ and presumed $CD8^+$ T cells. (**b**) Shown are dot plots for $CD4^+$ and presumed $CD8^+$ T cells of spleens harvested on day 13 representative of two mice per treatment arm.

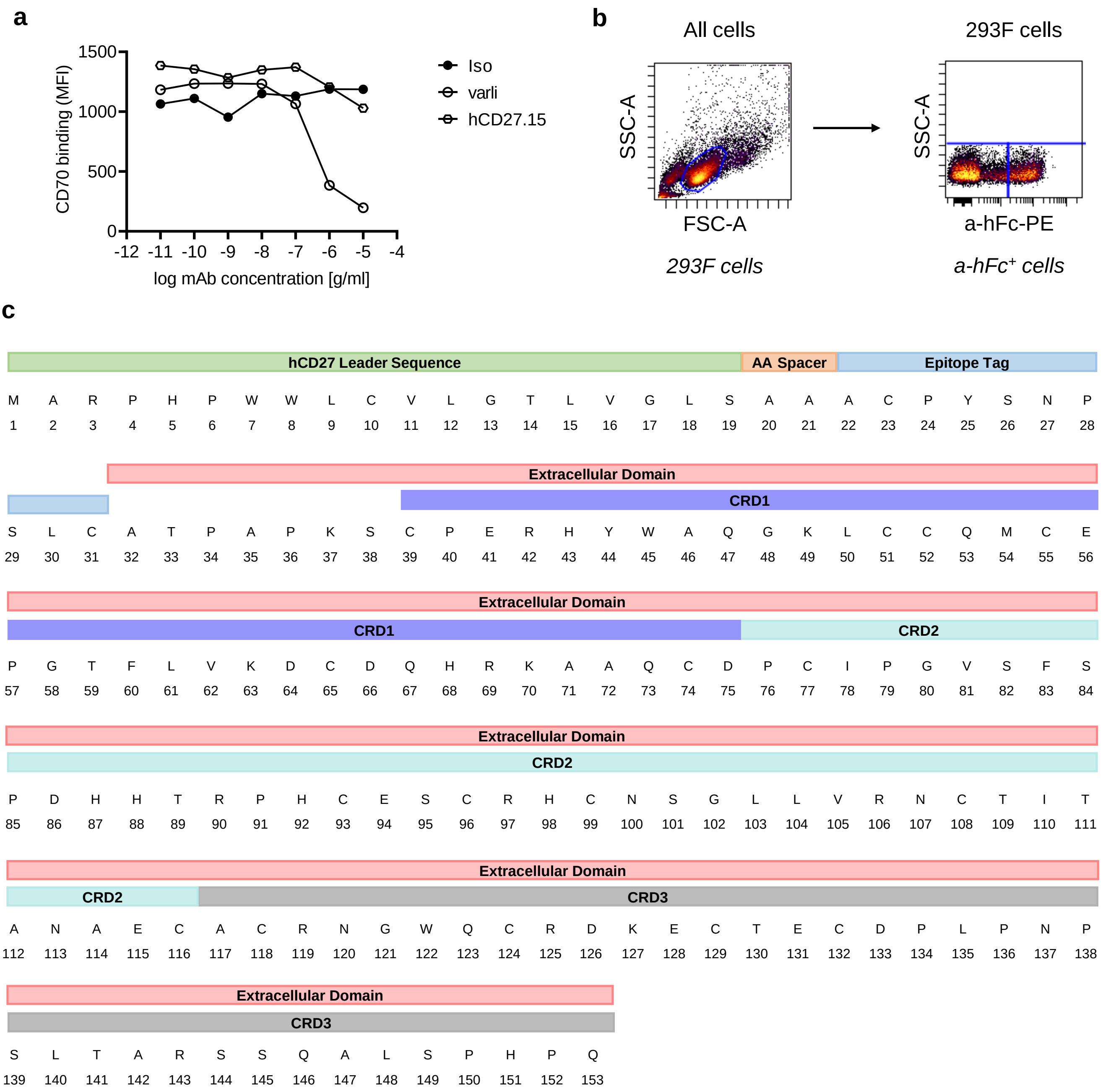

Supplementary Figure 4. CD70 competition analysis and epitope mapping of hCD27 mAb. a Human PBMC were incubated with either CD27.15 m1 or varli m1 or the irrelevant isotype control at the specified concentrations followed by CD70 h1 at 0.5 µg/ml. CD70 binding on CD4^{+} T cells was detected by flow cytometry using R-phycoerythrin-conjugated anti-hFc. The graph displays the binding of CD70 to the hCD27 receptor (MFI) in the presence of the respective hCD27 mAb. **b** 293F cells were transiently transfected with mutated hCD27 constructs and incubated with hCD27 mAb. Binding of the mAb to the constructs was assessed using R-phycoerythrin-conjugated anti-hFc by flow cytometry. The dot plots depict the gating strategy used to gate on hFc^{+} 293F cells. **c** hCD27 sequence (annotated with epitope tag) containing hCD27 leader sequence, hCD20 epitope tag and extracellular domains CRD1, CRD2 and CRD3. Numbers indicate the according amino acid position within the hCD27 sequence.

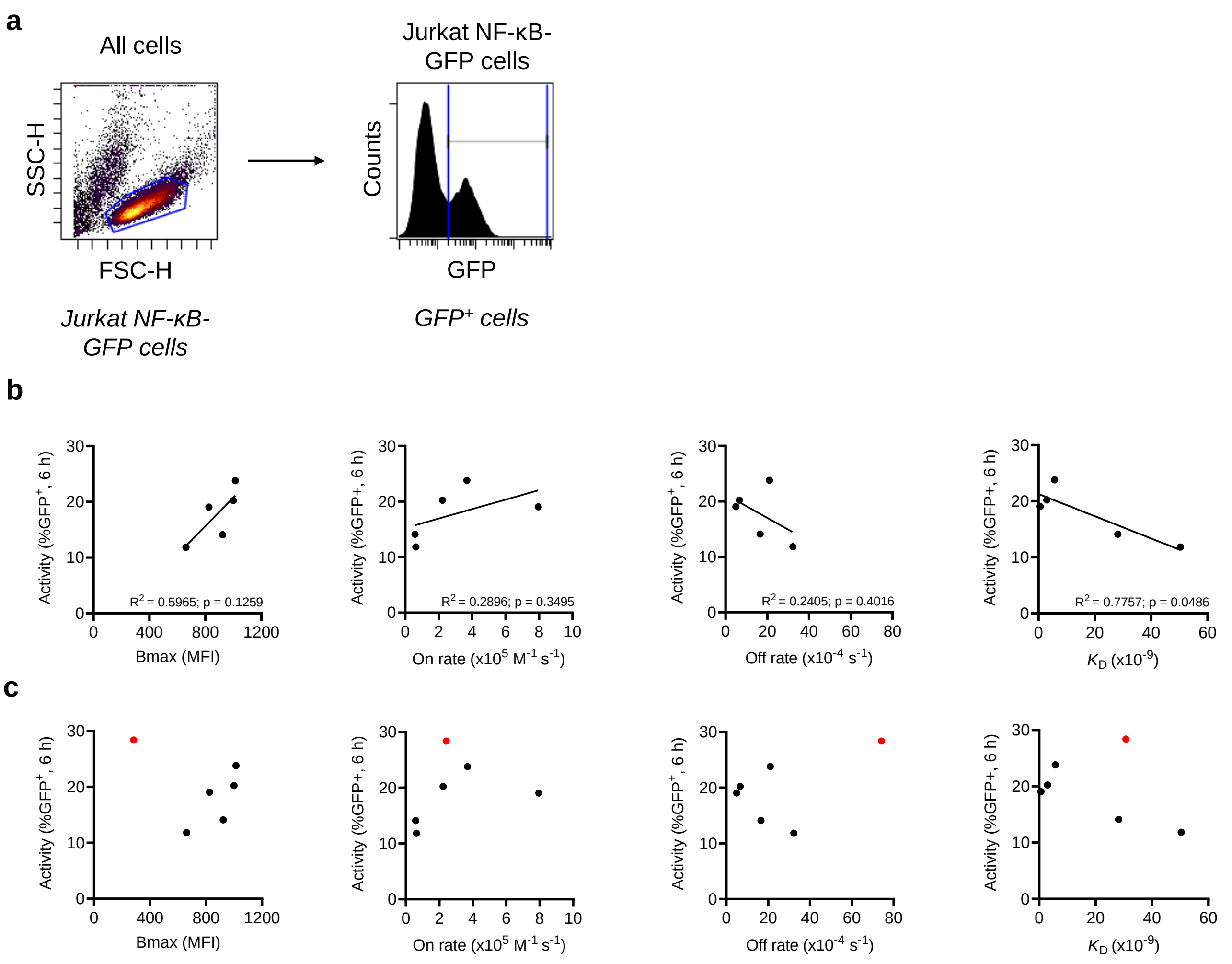

Supplementary Figure 5. NF-κB-GFP hCD27 Jurkat reporter assays. a All experiments involving hCD27 transfected NF-κB-GFP Jurkat cells were gated according to the strategy depicted in this figure. **b** NF-κB transcriptional activity of varli, AT133-2, AT133-5, AT133-11 and AT133-14 was plotted against K_D, Bmax, on rate and off rate. A linear regression was applied and data were assessed by Pearson correlation; *p <0.05. **c** NF-κB transcriptional activity of all hCD27 mAb, including hCD27.15 (coloured in red) was plotted against K_D, Bmax, on rate and off rate.

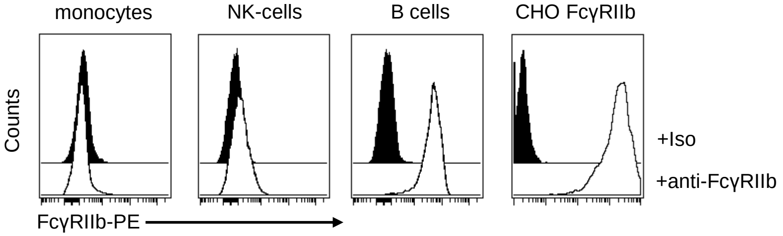

Supplementary Figure 6. FcγRIIb expression on immune cell subsets and FcγRIIb-transfected CHO cells. Expression of FcγRIIb on PBMC-derived monocytes, NK cells and B cells from healthy donors and on FcγRIIb-transfected CHO cells. Histograms are representative of n=2-3 experimental repeats.

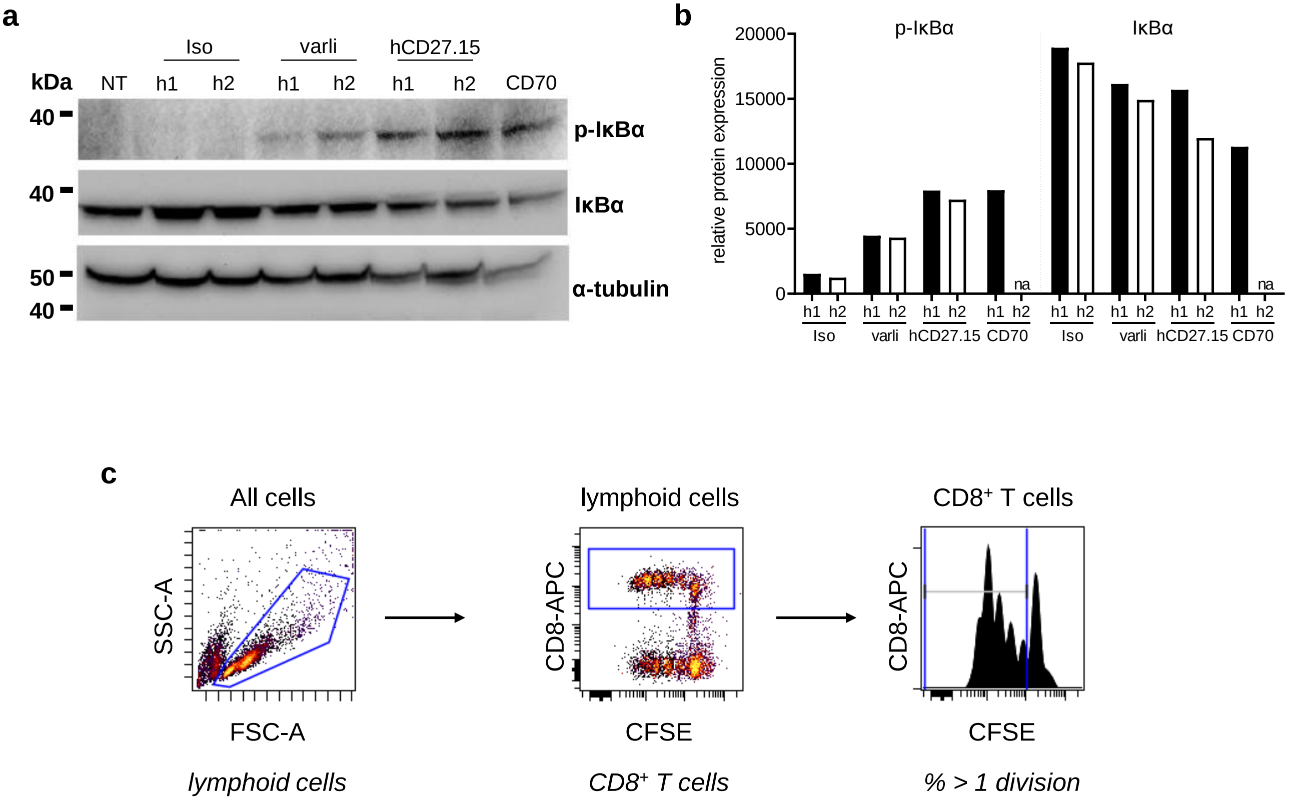

Supplementary Figure 7. Downstream signalling and T-cell proliferation upon stimulation with hCD27 mAb. **a, b** Jurkat cells expressing WT hCD27 were stimulated with the indicated hCD27 mAb (10 μg/ml), CD70 h1 (10 μg/ml) or the irrelevant control for 5 min and downstream signalling via the canonical NF-κB pathway was assessed by investigating expression of IκBα (39 kDa) and p-IκBα (40 kDa) by western blotting. (**a**) Expression of p-IκBα, IκBα and α-tubulin. (**b**) Relative protein expression analysed by densitometry using ImageJ software. Data representative of one experiment and graph shows mean. **c** Human PBMC were labelled with CFSE and incubated with hCD27 mAb for 4 days. CFSE dilution indicating T-cell proliferation was assessed by flow cytometry. Shown is the gating strategy for the %CD8$^+$ T cells that have undergone more than one division.

Supplementary Table 1. Residues defined as either active (antibody hypervariable loops) or passive (hCD27 residues) for information driven docking.

Complex	Fv - active residues	CD27 - passive residues
CD27 - varli	26, 27, 28, 29, 30, 31, 32, 53, 54, 55, 56, 100, 101, 102, 103, 104, 105, 106, 107, 145, 146, 147, 148, 149, 150, 151, 169, 170, 171, 210, 211, 212, 213, 214, 215	R97
CD27 – hCD27.15	26, 27, 28, 29, 30, 31, 32, 53, 54, 55, 56, 100, 101, 102, 103, 104, 142, 143, 144, 145, 146, 147, 148, 166, 167, 168, 207, 208, 209, 210, 211, 212	K63,D75
CD27 - AT133-2	25, 26, 27, 28, 29, 30, 31, 52, 53, 54, 55, 99, 100, 101, 102, 103, 104, 105, 106, 107, 145, 146, 147, 148, 149, 150, 151, 169, 170, 171, 210, 211, 212, 213, 214, 215	H68,R69
CD27 - AT133-5	26, 27, 28, 29, 30, 31, 32, 53, 54, 55, 56, 100, 101, 102, 103 ,104, 105, 143, 144, 145, 146, 147, 148, 149, 167, 168, 169, 208, 209, 210, 211, 212, 213	W45,K49
CD27 - AT133-11	26, 27, 28, 29, 30, 31, 32, 53, 54, 55, 56, 100, 101, 102, 103, 104, 105, 143, 144, 145, 146, 147, 148, 149, 150, 151, 152, 153, 171, 172, 173, 212, 213, 214, 215, 216, 217	W45,Q47, G48,K49
CD27 - AT133-14	25, 26, 27, 28, 29, 30, 31, 52, 53, 54, 55, 99, 100, 101, 102, 103, 104, 105, 106, 144, 145, 146, 147, 148, 149, 150, 151, 152, 153, 154, 172, 173, 174, 213, 214, 215, 216, 217, 218	D126,K127

Supplementary Table 2. Parameters of the best scoring models from the docking of hCD27 with the Fv portions of the hCD27 mAb of interest. Scores are relative to each system and cannot be compared between systems.

Complex	CD27 - Varli	CD27-hCD27.15	CD27-AT133-2	CD27-AT133-5	CD27-AT133-11	CD27-AT133-14
HADDOCK score	-65.3 +/- 1.3	-81.0 +/- 0.8	-83.9 +/- 1.1	-68.0 +/- 18.2	-74.0 +/- 4.2	-49.3 +/- 6.3
Cluster size	73	160	148	5	58	11
RMSD from overall lowest-energy structure	20.4 +/- 0.3	17.7 +/- 0.1	1.6 +/- 1.3	1.8 +/- 1.3	1.4 +/- 0.8	1.1 +/- 0.7
VdW energy	-50.0 +/- 6.0	47.7 +/- 4.9	-38.0 +/- 2.8	-39.6 +/- 6.6	-39.5 +/- 6.8	-32.5 +/- 8.0
Electrostatic energy	-181.9 +/- 27.6	-265.0 +/- 19.5	-390.4 +/- 29.2	-245.1 +/- 87.3	-308.6 +/- 11.2	-247.7 +/- 28.8
Desolvation energy	18.4 +/- 5.2	-20.3 +/- 2.6	-5.9 +/- 3.0	-8.4 +/- 1.6	-2.2 +/- 4.2	-1.0 +/- 2.0
Restraints violation energy	393.8 +/- 30.4	399.1 +/- 60.4	380.8 +/- 37.2	290.8 +/- 37.0	294.5 +/- 111.3	337.3 +/- 91.0
Buried surface area	1618.2 +/- 24.0	1639.4 +/- 43.6	1437.7 +/- 92.7	1298.0 +/- 37.0	1492.0 +/- 72.0	1237.0 +/- 48.9
Z-score	-1.1	-0.9	-1.0	-0.8	-1.5	-2.4
