# Supplementary material for: Agonistic CD27 antibody potency is determined by epitope-dependent receptor clustering augmented through Fc-engineering
Source: Commun Biol. 2022 Mar 14;5:229. doi: 10.1038/s42003-022-03182-6 (PMC8921514; doi:10.1038/s42003-022-03182-6)
Supplement: Supplementary file 5 — Reporting Summary [file 42003_2022_3182_MOESM5_ESM.pdf]

nature portfolio

Corresponding author(s): Sean Hua Lim

Last updated by author(s): Feb 9, 2022

# Reporting Summary

Nature Portfolio wishes to improve the reproducibility of the work that we publish. This form provides structure for consistency and transparency in reporting. For further information on Nature Portfolio policies, see our Editorial Policies and the Editorial Policy Checklist.

## Statistics

For all statistical analyses, confirm that the following items are present in the figure legend, table legend, main text, or Methods section.

| n/a | Confirmed | |
|---|---|---|
| ☐ | ☒ | The exact sample size ($n$) for each experimental group/condition, given as a discrete number and unit of measurement |
| ☐ | ☒ | A statement on whether measurements were taken from distinct samples or whether the same sample was measured repeatedly |
| ☐ | ☒ | The statistical test(s) used AND whether they are one- or two-sided<br>*Only common tests should be described solely by name; describe more complex techniques in the Methods section.* |
| ☐ | ☒ | A description of all covariates tested |
| ☐ | ☒ | A description of any assumptions or corrections, such as tests of normality and adjustment for multiple comparisons |
| ☐ | ☒ | A full description of the statistical parameters including central tendency (e.g. means) or other basic estimates (e.g. regression coefficient) AND variation (e.g. standard deviation) or associated estimates of uncertainty (e.g. confidence intervals) |
| ☒ | ☐ | For null hypothesis testing, the test statistic (e.g. $F$, $t$, $r$) with confidence intervals, effect sizes, degrees of freedom and $P$ value noted<br>*Give P values as exact values whenever suitable.* |
| ☒ | ☐ | For Bayesian analysis, information on the choice of priors and Markov chain Monte Carlo settings |
| ☒ | ☐ | For hierarchical and complex designs, identification of the appropriate level for tests and full reporting of outcomes |
| ☒ | ☐ | Estimates of effect sizes (e.g. Cohen's $d$, Pearson's $r$), indicating how they were calculated |

*Our web collection on statistics for biologists contains articles on many of the points above.*

## Software and code

Policy information about availability of computer code

| | |
|---|---|
| Data collection | FACS samples were acquired on BD FACS Canto II or BD FACS Calibur software. Biacore data was collected using Biacore T200 Evaluation software (GE Healthcare Life Sciences, RRID: SCR_003070) and microscopy images were aquired using NimOS 1.18 software (ONI Oxford). Western blot images were aquired using Biospectrum AC Imaging system software. |
| Data analysis | FACS data: Cytobank 7.2 software (RRID: SCR_014043), Images (microscopy/western blot): Image J 1.52 (RRID: SCR_019718), Statistics: Graphpad Prism 9 (RRID: SCR_002798) |

For manuscripts utilizing custom algorithms or software that are central to the research but not yet described in published literature, software must be made available to editors and reviewers. We strongly encourage code deposition in a community repository (e.g. GitHub). See the Nature Portfolio guidelines for submitting code & software for further information.

## Data

Policy information about availability of data

All manuscripts must include a data availability statement. This statement should provide the following information, where applicable:

- Accession codes, unique identifiers, or web links for publicly available datasets
- A description of any restrictions on data availability
- For clinical datasets or third party data, please ensure that the statement adheres to our policy

All data supporting the findings of this study are available within the published article and its supplementary information files. Source data are provided in Supplementary Data 1. All other data are available from the corresponding author upon reasonable request.

# Field-specific reporting

Please select the one below that is the best fit for your research. If you are not sure, read the appropriate sections before making your selection.

☒ Life sciences ☐ Behavioural & social sciences ☐ Ecological, evolutionary & environmental sciences

For a reference copy of the document with all sections, see nature.com/documents/nr-reporting-summary-flat.pdf

# Life sciences study design

All studies must disclose on these points even when the disclosure is negative.

| | |
|---|---|
| Sample size | No statistical methods were used to predetermine sample size in either in vivo/ex vivo or in vitro experiments. For in vivo/ex vivo experiments sample size were chosen based on previous experience in the mouse models used (PMID: 29198913). For experiments involving animals, at least n=4 mice per group were used.<br>For in vitro studies, the experiments were repeated at least 3 times (with exception of confocal microscopy, western blot and cross-blocking of CD70 with varli and hCD27.15) with samples size of at least 3 within each experiment to control technical variations. For the experiments that using human PBMC, experiments were repeated with at least 3 different donors to control for biological variations. |
| Data exclusions | No data were excluded from the analyses. |
| Replication | Experiments including animals were repeated at least one time as stated in the figure legends. Due to low variance, the animal experiment assessing the difference between anti-CD27 m1 and m2a as well as the difference between hCD27.15 m1 and h2 was only conducted once, and statistical power was achieved with the group size employed.<br>For in vitro studies (except microscopy, western blot and cross-blocking of CD70 with varli and hCD27.15) at least 3 independent experiments were performed. Microscopy was only conducted once, since the number of cells assessed showed low variance and statistical power was achieved with the cell numbers assessed. The western blot and cross-blocking assay was only conducted once since both experiments were merely used as supporting data and confirmation for already existing data.<br>Details of experimental replicates are stated in the figure legends. All attempts at replication were successful. |
| Randomization | Randomization of mice into treatment groups was performed. Prior to treatment, we ensured that the mean age were comparable among the various treatment groups. No randomization was used for the in vitro experiments, as it is not applicable to the study design. |
| Blinding | For the microscopy experiment, the evaluation of cluster size and numbers were collected by a blinded researcher. For the animal survival experiments, the mice were assessed by blinded researchers. In all other experiments, the investigators were not blinded. |

# Reporting for specific materials, systems and methods

We require information from authors about some types of materials, experimental systems and methods used in many studies. Here, indicate whether each material, system or method listed is relevant to your study. If you are not sure if a list item applies to your research, read the appropriate section before selecting a response.

## Materials & experimental systems

| n/a | Involved in the study | |
|---|---|---|
| ☐ | ☒ | Antibodies |
| ☐ | ☒ | Eukaryotic cell lines |
| ☒ | ☐ | Palaeontology and archaeology |
| ☐ | ☒ | Animals and other organisms |
| ☒ | ☐ | Human research participants |
| ☒ | ☐ | Clinical data |
| ☒ | ☐ | Dual use research of concern |

## Methods

| n/a | Involved in the study | |
|---|---|---|
| ☒ | ☐ | ChIP-seq |
| ☐ | ☒ | Flow cytometry |
| ☒ | ☐ | MRI-based neuroimaging |

## Antibodies

| | |
|---|---|
| Antibodies used | hCD27 hIgG1 (varlilumab)/hIgG2 In house based on sequence information of Keler et al. (Keler et al., 2015)<br>hCD27.15 hIgG1/hIgG2/hIgG1 V11/hIgG1 SELF In-house based on sequence information of Van Eenennaam et al. (Van Eenennaam et al., 2016)<br>hCD27 hIgG1/hIgG2/hIgG1 V11/hIgG1 SELF In-house Clone: AT133-2, AT133-5, AT133-11, AT133-14<br>Anti-CD27 mIgG1/mIgG2a In-house Clone: AT124-1<br>mCTLA-4 (9D9) BioXCell Cat. # BE0164; RRID: AB_10949609<br>mCD25 (PC61) In-house produced from hybridoma purchased from ATCC PC61 hybridoma cells: ATCC TIB-222; RRID: AB_2125456<br>mCD20 (18B12) In-house based on sequence information of Dunn et al. (Dunn et al., 2007); Clone: 18B12<br>PE-labelled goat anti-human IgG Fc secondary antibody Jackson Immunoresearch Cat # 109-116-170; RRID: AB_2337681<br>Viability Dye eF506 eBioscience Cat. # 65-0866-14<br>mCD3 FITC (OKT3) In-house produced from hybridoma purchased from ATCC OKT3 hybridoma cells: ATCC® CRL-8001; RRID: |

AB_2073169
mCD4 eF450 (GK1.5) eBioscience Cat. # 48-0041-82; RRID: AB_10718983
mCD25 APC (PC61.5) eBioscience Cat. # 17-0251-82; RRID: AB_469366
mFoxP3 PE (NRRF-30) eBioscience Cat. # 12-4771-82; RRID: AB_529580
mCD45 PerCP Cy5.5 (30-F11) eBioscience Cat. # 45-0451-82; RRID: AB_1107002
mF4/80 Alexa 647 (CI:A3-1) Serotec/BioRad Cat. # MCA497A64; RRID: AB_323931
mLy6c APC eF780 (HK1.4) eBioscience Cat. # 47-5932-82; RRID: AB_2573992
mLy6G PE (RB6-8C5) eBioscience Cat. # 12-5931-82; RRID: AB_466045
mCD11b eF450 (M1/70) eBioscience Cat. # 48-0112-82; RRID: AB_1582236
mCD8 APC eF780 (53-6.7) eBioscience Cat. # 47-0081-82; RRID: AB_1272185
mFcγRIV FITC (9E9 IgG) In-house (Tipton et al., 2015)
hCD8 APC (SK1) eBioscience Cat. # 17-0087-42; RRID: AB_1311204
hFcγRIIb PE (6G11) kind gift from BioInvent International N/A
hCD3 FITC (SK7) eBioscience Cat. # 11-0036-42; RRID: AB_1272072
hCD56 APC (N901) BD Cat # IM2474
hCD19 PerCP eF710 (J3-129) eBioscience Cat. # 46-0197-42, RRID: AB_10669;10
hCD14 Pacific Blue (M5E2) BioLegend Cat. # 982504; RRID: AB_2632615
hCD4 PerCP Cy5.5 (RPA-T4) eBioscience Cat. # 45-0049-42; RRID: AB_1518744
hCD3 (OKT3) in-house
p-IκBα (Ser32/36) (5A5) Cell Signaling Cat # 9246; RRID: AB_2267145
IκBα (Ser32) (14D4) Cell Signaling Cat # 2859; RRID: AB_561111
α-tubulin (11H10) Cell Signaling Cat # 2125; RRID: AB_2619646

Validation

Antibodies produced in-house were validated by ELISA. Commercially available and validated antibodies were validated on known cell subsets.

## Eukaryotic cell lines

Policy information about cell lines

Cell line source(s)

Mouse: BCL1 In-house (French et al., 2007)
Mouse: CT26 cells ATCC ATCC CRL-263; RRID: CVCL_7256
Freestyle 293 F cells ThermoFisher Scientific Cat # R79007; RRID: CVCL_D603
CHO-K1 ATCC ATCC CCL-61; RRID: CVCL_021
FcγRIIb CHO-K1 In-house (Tutt et al., 2015)
NF-κB/GFP hCD27 Jurkat cells NF-κB Jurkat GFP purchased from System Biosciences, transfected in house Cat # TR850A-1 (NF- κB Jurkat GFP cells)
hCD27 Jurkat Parental Jurkat cells purchased from ATCC, transfected in-house ATCC TIB-152; RRID: CVCL_0367 (Jurkat E6-1 cells)
hCD27/GFP Jurkat Parental Jurkat cells purchased from ATCC, transfected in-house ATCC TIB-152; RRID: CVCL_0367 (Jurkat E6-1 cells)

Authentication

Cell lines did not undergo complete authentication upon receipt from the source indicated. Cell lines obtained from sources such as ATCC were authenticated prior to purchase and rapidly expanded in culture and cryopreserved at early stages. Parental cell lines stably transfected with antigens of interest were regularly tested for antigen-expression by flow cytometry.

Mycoplasma contamination

The cell lines were tested mycoplasma-free

Commonly misidentified lines
(See ICLAC register)

Cell lines employed were not listed in the misidentified cell lines database curated by the ICLAC

## Animals and other organisms

Policy information about studies involving animals; ARRIVE guidelines recommended for reporting animal research

Laboratory animals

Mouse: BALB/c Purchased from Charles River, maintained in house Strain Code: 028
Mouse: hCD27 tg: BALB/c.B6-Tg(hCD27) BALB/c Celldex Therapeutics (He et al., 2013)
Mouse: FcγRIV-/-BALB/c In house (Tipton et al., 2015)

All mice were fed regular chow, had water freely and were maintained in a conventional facility. All experiments were conducted with age-matched (8-12 week old) female mice. Animals were randomly assigned to experimental groups and housed together under the same conditions.

Wild animals

The study did not involve wild animals

Field-collected samples

The study did not involve field-collected samples

Ethics oversight

All experiments were conducted according to the UK Home Office license guidelines and in accordance with the Animals (Scientific Procedures) Act 1986 under the Procedure Project Licences P81E129B7 and P4D9C89EA and approved by the University of Southampton Ethical Committee.

Note that full information on the approval of the study protocol must also be provided in the manuscript.

# Flow Cytometry

## Plots

Confirm that:

☒ The axis labels state the marker and fluorochrome used (e.g. CD4-FITC).

☒ The axis scales are clearly visible. Include numbers along axes only for bottom left plot of group (a 'group' is an analysis of identical markers).

☒ All plots are contour plots with outliers or pseudocolor plots.

☒ A numerical value for number of cells or percentage (with statistics) is provided.

## Methodology

| | |
|---|---|
| Sample preparation | For flow cytometric analysis single cell suspensions were generated. Murine tissue samples were firstly incubated with Fc-gamma receptor block (where the antigens of interest were not FcgRs), following incubation with antibodies for specific markers of interest and subsequently underwent red blood cell lysis and fixation in 2% paraformaldehyde and further processed as outlined in the methods. For intracellular staining, cells were fixed and permeabilized and stained with the antibodies against intracellular markers of interest. For enumeration and analysis of myeloid cells, tissue was chopped into small pieces and digested using Liberase TL (Sigma Aldrich) according to the manufacturer's protocol. Digested tissue was then mashed into a single cell suspension and treated as outlined above. |
| Instrument | Samples were analyzed on a BD FACSCanto II or BD FACSCalibur (BD Biosciences) |
| Software | Data were collected using FACSDiva software (BD Pharmingen) and analyzed using Cytobank software version 7.2 |
| Cell population abundance | No cell sorting was performed. |
| Gating strategy | Gating strategies for all relevant experiments are depicted in the manuscript. |

☒ Tick this box to confirm that a figure exemplifying the gating strategy is provided in the Supplementary Information.
